# Biogeographic, Driving Factors, Assembly, and Co-occurrence Patterns of Archaeal Community in Biocrusts

**DOI:** 10.3389/fmicb.2022.848908

**Published:** 2022-04-12

**Authors:** Yuanlong Li, Jingyi Wei, Haijian Yang, Delu Zhang, Chunxiang Hu

**Affiliations:** ^1^Key Laboratory of Algal Biology, Institute of Hydrobiology, Chinese Academy of Sciences, Wuhan, China; ^2^University of Chinese Academy of Sciences, Beijing, China

**Keywords:** archaeal community, biocrusts, biogeographic pattern, assembly, co-occurrence

## Abstract

Archaea exhibit strong community heterogeneity with microhabitat gradients and are a non-negligible part of biocrust’s microorganisms. The study on archaeal biogeography in biocrusts could provide new insights for its application in environmental restoration. However, only a few studies on assembly processes and co-occurrence patterns of the archaeal community in patchy biocrusts have been reported, especially considering the number of species pools (SPs). Here, we comprehensively collected biocrusts across 3,500 km of northern China. Different successional biocrusts from various regions contain information of local climate and microenvironments, which can shape multiple unique archaeal SPs. The archaeal community differences in the same successional stage exceeded the variations between successional stages, which was due to the fact that the heterogeneous taxa tended to exchange between unknown patches driven by drift. We also comparatively studied the driving forces of community heterogeneity across three to ten SPs, and assembly and co-occurrence patterns were systematically analyzed. The results revealed that the impact of spatial factors on biogeographic patterns was greater than that of environmental and successional factors and that impact decreased with the number of SPs considered. Meanwhile, community heterogeneity at the phylogenetic facet was more sensitive to these driving factors than the taxonomic facet. Subgroups 1 (SG1) and 2 (SG2) of the archaeal communities in biocrusts were dominated by Nitrososphaeraceae and Haloarchaea, respectively. The former distribution pattern was associated with non-salinity-related variables and primarily assembled by drift, whereas the latter was associated with salinity-related variables and primarily assembled by homogeneous selection. Finally, network analysis indicated that the SG1 network had a higher proportion of competition and key taxa than the SG2 network, but the network of SG2 was more complex. Our study suggested that the development of the archaeal community was not consistent with biocrusts succession. The dominant taxa may determine the patterns of community biogeography, assembly, and co-occurrence.

## Introduction

Archaea are an important microbial community component in the tree of life, which has essential ecological roles. It widely inhabits extreme mesophilic environments. Until present, approximately 25% of archaea are being discovered on the land surface (Flemming and Wuertz, 2019), which are crucial for nutrient management, crop productivity, and biofilm formation (Shi et al., 2018; van Wolferen et al., 2018). The mechanisms that regulate the archaeal community biogeography, composition, assembly, and co-occurrence patterns are fundamental to microbial ecology but have rarely been studied at the oxygen-light interface in dryland, where biocrusts are abundant. Furthermore, rising temperatures are projected to affect nutrient loading and evaporation in current global climate models (Rautio et al., 2011), making the dryland ecosystems even more fragile. Therefore, there is an urgent need to provide knowledge of archaeal biogeographic patterns in dryland topsoil at a large spatial scale.

Even within small environmental changes, archaeal communities exhibit high heterogeneity, as demonstrated in oceans (Luo et al., 2015; Zhang and Li, 2020), rivers (Liu et al., 2018), hot springs (Narsing Rao et al., 2021), and sediments (Li and Gu, 2013; Zou et al., 2020), emphasizing that biogeographic patterns are influenced by environmental factors, such as salinity, electrical conductance (EC) (Hollister et al., 2010; Pandit et al., 2015), pH (Tripathi et al., 2015), nutrient availability (Bates et al., 2011), and macroclimate (Zhang J. et al., 2018). Moreover, the impact of unmeasured spatial factors cannot be disregarded, particularly at large spatial scales (Jiao et al., 2019; Ladau and Eloe-Fadrosh, 2019; Picazo et al., 2020). However, spatial and environmental factors frequently change synchronously (Wang K. et al., 2019), driving community variation at both taxonomic and phylogenetic facets (Martiny et al., 2011). Therefore, the results of archaeal biogeographic patterns depended not only on transect scales designed (Mestre et al., 2017) but also on a comparison of spatial and environmental driving forces. Furthermore, there should be more isolated species pools (SPs) due to the susceptible community heterogeneity of archaea, which has also altered biogeographic patterns (Wang et al., 2013b; Fukami, 2015). In addition, community heterogeneity converging along with succession (Ortiz-Alvarez et al., 2018; Xu et al., 2020; Gao et al., 2021) was another influencing factor while remaining unclear in archaea. Therefore, a comprehensive study incorporating these factors needs to be conducted urgently.

Understanding the community assembly processes is a major objective of microbial ecology, frequently employing a quantitative framework based on phylogenetic signals (Stegen et al., 2013; Wang et al., 2013a; Dini-Andreote et al., 2015; Tripathi et al., 2018; Jiao et al., 2020; Li and Hu, 2021). Results compared the influence of stochastic and deterministic processes in various habitats (Logares et al., 2018; Mo et al., 2018; Wu et al., 2018), successional stages, and meridional gradients (Ortiz-Alvarez et al., 2018; Li and Hu, 2021). The possible explanations were mostly at the community level with inconspicuous community structure characteristics and were attributed to habitats with diverse phylogenesis and specific abiotic factors (Dini-Andreote et al., 2015; Tripathi et al., 2018; Li and Hu, 2021). Recent studies have displayed the relationships between co-occurrence patterns based on network topological features and community assembly processes (Jiao et al., 2020), emphasizing a key taxa role in maintaining network persistence and participating in the assembly (Banerjee et al., 2018). However, few studies have been conducted on biocrusts located at the oxygen-light interface, which provides a novel habitat to be studied and could generate new community assembly theories. Although the investigations of bacterial and eukaryotic community assembly in biocrusts have been conducted (Li and Hu, 2021), the assembly knowledge of the archaeal community remains limited. Given the conspicuous characteristics of archaeal community structure, dominant taxa’s survival strategies could reveal new insights into the assembly.

Biocrusts are a common formation at the topsoil layer, which accounts for 12% of the Earth’s land surface (Weber et al., 2016). Biocrusts are considered a model ecosystem for the study of microbial communities due to their distinct successional stages, which are classified as algal (A), Cyanobacterial lichen (C), and moss (M) crust based on cryptogamous abundance (Wu et al., 2014). This dynamic succession process benefits the studies of biogeographic, assembly, and co-occurrence patterns in microbial ecology (Meiners et al., 2015). Therefore, we conducted a comprehensive sampling across three climatic zones in northern China spanning 3,500 km and collected 200 biocrusts containing 140 A, 24 C, and 36 M crusts. The archaeal community was analyzed using high-throughput screening, concomitantly with measurements of physicochemical properties, extracellular enzyme activity and macroclimate parameters. The driving factors of successional, spatial, and environmental on community variation were compared at both taxonomic and phylogenetic facets under various numbers of SPs. Furthermore, the biogeographic, assembly, and co-occurrence patterns from different phylogenetic taxa were comparatively studied. These analyses enabled us to address the following questions: (i) What are the most important successional, geographical, and environmental driving factors for the differences in archaeal communities? (ii) What are the biogeographic patterns of archaea under these factors’ influence and how do the impacts vary with the SPs? (iii) What is the difference between assembly and co-occurrence patterns from different phylogenetic archaeal taxa?

## Materials and Methods

### Soil Sampling

**FIGURE 1:**
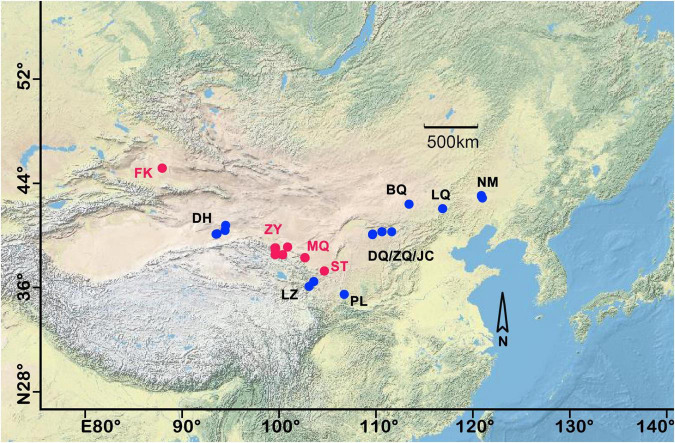
Study area and sampling plots of north China. Each sample plot was named by the abbreviation of the place name. The red dots indicated that three different successional stages of biocrust were synchronously collected. FK, Fukang; DH, Dunhuang; ZY, Zhangye; MQ, Minqin; LZ, Lanzhou, ST, Shapotou, PL, Pingliang, DQ, Datela Qi; ZQ, Zhungeer Qi; JC, Jiechai; BQ, Bai Qi; LQ, Lan Qi; NM, Naiman. For more detailed sample information, refer to our previous research (Li and Hu, 2021).

A comprehensive sampling of biocrusts was collected across seven major deserts and 3,500 km in northern China. A total of 200 samples were collected from 13 plots, including 140 A, 24 C, and 36 M-dominated biocrusts. Each sample plot was considered an SP, as shown in Figure 1. Each sample plot had received no rainfall in the past 72 h and kept 0.2 m away from the shrubs. The biocrust sample covered by a single biotype was selected for each successional stage. The biocrusts and attached subsoil were gathered with a shovel and preserved in sterilized plastic Petri dishes to ensure the integrality and then transported to the laboratory within 12 h. To ensure the non-redundancy and representativeness of the sequencing results, the macroscopic moss plants (but not their protonemata) were removed from M biocrusts (Li and Hu, 2021).

### Amplicon Sequencing and Environmental Data Collection

Total genomic DNA was extracted from biocrust samples using PowerSoil^®^ DNA Isolation Kit (MOBIO, United States). 16S rDNA (524F10extF/Arch958RmodR, V4–V5 region) primers were used for amplification. Sequencing was performed on the Illumina MiSeq PE300 platform (Illumina, San Diego, CA, United States). The raw sequence data were uploaded on NCBI under BioProject PRJNA730649. The acquired sequences were filtered for quality control following standard procedures (Shanghai Majorbio Bio-Pharm Technology Co., Ltd.). The operational taxonomic unit (OTU) was generated from defined representative sequences, which set clustering at 97% similarity. Ribosomal database project (RDP) classifier was used for taxonomic annotation of representative sequences based on an identity threshold of 0.7 in the SILVA 132 database for archaea (16S_archaea).

Microenvironments included water content (WC), soil texture (%), thickness (TH., mm), chlorophyll *a* (Chl *a*, μg⋅g^–1^), scytonemin (Scyt., unit⋅mg^–1^ FW), extracellular polysaccharide (EPS, mg⋅g^–1^), bacteriochlorophyll *a* (BChl *a*, μg⋅g^–1^), variable fluorescence/maximal fluorescence (Fv/Fm), pH, ORP, salinity (μmol⋅g^–1^), NH_4_^+^ (μmol⋅g^–1^), PO_4_^3–^ (μmol⋅g^–1^), total nitrogen (TN, g⋅kg^–1^), total phosphorus (TP, g⋅kg^–1^), total organic carbon (TOC, g⋅kg^–1^), soil alkaline protease (ALPT), soil-β-glucosidase (β-GC), and soil alkaline phosphatase (ALP). Macroclimates included mean annual precipitation (MAP), aridity index (AI), mean annual sunshine duration (MASD), mean annual temperature (MAT), altitude (Alt.), and wind speed (WS). These variables were determined by previous methods (Li and Hu, 2021).

### Statistical Analysis

To study the driving forces on the difference of archaeal communities varied with the number of SPs, the combined meta-community dataset containing the different number of sample plots was derived by the method of combination. First, there were 13 sample plots, and each was considered an SP. We calculated ${\text{C}}_{13}^{3}$, ${\text{C}}_{13}^{4}$, ${\text{C}}_{13}^{5}$, ${\text{C}}_{13}^{6}$, ${\text{C}}_{13}^{7}$, ${\text{C}}_{13}^{8}$, ${\text{C}}_{13}^{9}$, and ${\text{C}}_{13}^{10}$, corresponding to three to ten SPs. Each combined dataset included at least one of the Fukang, Zhangye, Minqin, and Shapotou sample plots and all three successional biocrusts (Figure 1). 202, 589, 1161, 1632, 1680, 1278, 714 and 286 combined communities were generated under the three to ten SPs respectively. This step enabled us to measure the influence of successional factors on the variation of combined meta-communities. Second, the permutational multivariate analysis of variance (PERMANOVA, permutation = 999) was used to calculate the explanations (*R*^2^) of successional stages (i.e., successional factor), geographical locations (i.e., spatial factor), and each environmental variable (i.e., environmental factor) for each combined meta-community variation-based Bray–Curtis and UniFrac-Weighted dissimilarity. Third, the geographic distance of a combined meta-community dataset was defined by the distance between the farthest sample plots in the given dataset. Subsequently, the linear regression was conducted between the explanation of these three factors and geographic distance (*<0.05, ^**^<0.01), and the slope was used to indicate the driving forces of the three factors affecting the biogeographic patterns. Note that the explanation of environmental factors for the given combined meta-community was represented by a weighted mean explanation of all environmental factors to avoid Simpson’s Paradox error. The phylogenetic tree was constructed by IQ-TREE (version 1.6.8^1^) based on the maximum likelihood method with default procedures and was used to calculate the UniFrac-Weighted dissimilarity.

To analyze the characteristics of archaeal community structure and its covariant relationship with environments, the redundancy analysis (RDA) was performed at a family level. Then, the forward selection procedure was used to screen out significant environmental variables (Blanchet et al., 2008). The analysis of similarities (ANOSIM) (permutation test = 999) was used to examine community differences among successional stages and between subgroups. Linear regression analysis (*p < 0.05, ^**^p < 0.01) was used to assess the goodness of fit (*R*^2^) for the first axis coordinates of non-metric multidimensional scaling (NMDS) with environmental factors (standardization) in each successional stage. To explore the correlation between community variation and environmental variables, a mantel test analysis was performed with community matrices at OTU level and environmental factors. ANOVA was used to analyze the significant differences of α and β diversity between three successional stages [least significant difference (LSD), *p < 0.05, ^**^p < 0.01]. All of the above results were visualized by box and whisker (10–90%). We referred to Stegen’s method (Stegen et al., 2013) to quantify the relative importance of ecological processes. β-Nearest-taxon-index (βNTI) values of >2 or <−2 indicated heterogeneous selection or homogeneous selection, respectively, whereas if the absolute values of βNTI were <2, stochastic processes would play an important role. Then, the Raup–Crick metric using Bray–Curtis dissimilarities (RC_bray_) values <−0.95 pointed to a community assembly governed by homogeneous dispersal. In contrast, dispersal limitation could generate RC_bray_ values >+0.95. Drift is only expected when RC_bray_ values were between −0.95 and +0.95. We inferred a co-occurrence network for the archaeal community at the OTU level using the default settings on the MENA website^2^ and generated characteristics of topological structure. We identified key and peripheral species in the network by the within-module connectivity (*Zi*) and among-module connectivity (*Pi*) based on Deng’s method (Deng et al., 2012).

The R environment (version 3.6.2^3^) was used for relevant statistical analyses.

## Results

### The Changes of Biodiversity and Assembly Processes of Archaeal Communities With Succession

**FIGURE 2:**
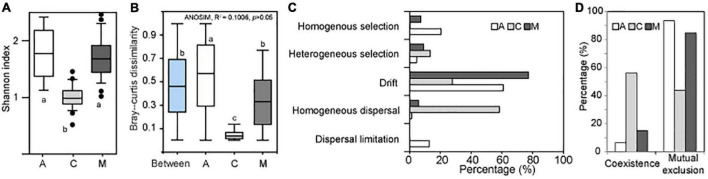
The changes of archaeal biodiversity and assembly processes with succession. The Shannon index **(A)** was calculated in algae (A), Cyanobacterial lichen (C), and moss-dominated (M) crusts. The paired Bray–Curtis dissimilarities **(B)** were, respectively, calculated in A, C, and M successional crusts. Besides, the paired Bray–Curtis dissimilarities between different successional stages were also calculated (i.e., Between). The community differences among three successional stages were studied by analysis of similarities (ANOSIM). A significant difference among three successional stages using analysis of variance (ANOVA; df = 2, least significant difference, small letters indicated *p* < 0.05). The assembly processes **(C)** of archaeal community in different successional stages were quantified using Stegen’s method (Stegen et al., 2013). The topological characteristics **(D)** of co-occurrence network were calculated on the MENA website (http://ieg4.rccc.ou.edu/mena).

The α diversity of archaeal communities in A and M crusts was significantly higher than that in C crusts (Figure 2A). The results of ANOSIM showed that there was no significant difference in the archaeal community among the three successional stages (Figure 2B). In contrast, in the same successional stage, the differences of archaeal communities in A and M crusts were larger than that in C crusts and the difference in A crusts has exceeded the difference between succession. The results of ecological processes showed that the archaeal communities were mainly assembled by drift in A and M crusts, while they were mainly dominated by homogenous dispersal in C crust (Figure 2C). The co-occurrence patterns demonstrated that mutual exclusion was dominant in A and M crusts, while coexistence was dominant in C crust (Figure 2D). In summary, the assemblages mainly governed by drift had higher α diversity, community differences, and mutual exclusion in A and M crusts than in C crusts.

### The Driving Forces of Spatial, Environmental, and Successional Factors

**FIGURE 3:**
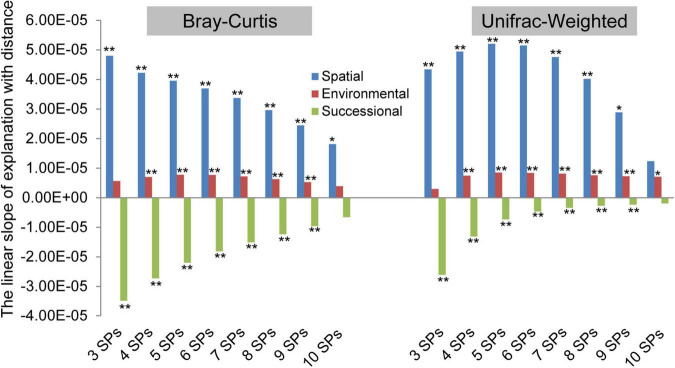
The driving forces of spatial, environmental, and successional factors on archaeal community differences varied with the number of species pools (SPs). The permutational multivariate analysis of variance (permutation = 999) was used to calculate the explanations (*R*^2^) of successional stages (i.e., successional factor), geographical locations (i.e., spatial factor), and each of the environmental variables (i.e., environmental factor) for each combined meta-community variation based Bray–Curtis and UniFrac-Weighted dissimilarity. The number of sample plots contained in the combined community was considered the number of SPs. The slope was calculated by linear regression (*<0.05, **<0.01) between the explanations (*R*^2^) and distance geographic distance (km), which was used to indicate the driving force of the three factors, affecting the biogeographic pattern.

The biogeographic patterns based on both taxonomic and phylogenetic variations, which are explained by successional, spatial, and environmental factors, were examined (Figure 3 and Supplementary Figure 1). In terms of slope direction, the explanation of spatial and environmental factors increased with distance, but successional factors decreased. At the taxonomic variation facet, the order of slope absolute value was spatial > successional > environmental, and the slope absolute value of spatial and successional factors decreased with the number of SPs. In addition, based on phylogenetic variation, the order was spatial > successional > environmental at three and four SPs. In contrast, with five to ten SPs, the order was spatial > environmental > successional, and the slope absolute value of spatial and successional order decreased. In summary, spatial and environmental factors were positive driving forces at both taxonomic and phylogenetic facets of archaeal community variation, while successional factors were negative in three to ten SPs. The impact of the spatial factor was the most significant, but it differed at both taxonomic and phylogenetic variation facets.

### Archaeal Community Structure and Distribution in Biocrusts

**FIGURE 4:**
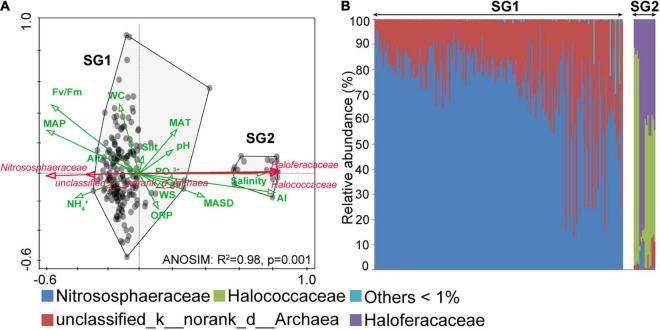
Redundancy analysis (RDA) ordination and archaeal community structures. **(A)** The RDA results showed the environmental variables, which were indicated as green arrows, and the dominant taxa were indicated as red arrows. The community differences between subgroup1 (SG1) and subgroup2 (SG2) were calculated by ANOSIM. **(B)** The 200 archaeal community structures were arranged in ascending order of diversity (Shannon index, operational taxonomic unit level). AI, aridity index; MAP, mean annual precipitation; WC: water content, ORP, oxidation-reduction potential; WS, wind speed; MAT, mean annual temperature; MASD, mean annual sunshine duration; Alt., altitude.

Analysis of similarities and RDA were, respectively, used to examine differences between the archaeal community structure and significant environments at the family level. The total variation of the archaeal community was explained most by AI, followed by salinity and PO_4_^3+^ (Supplementary Table 2). Two hundred samples were clustered into two subgroups: subgroup1 (SG1) and subgroup2 (SG2) (Figure 4A; ANOSIM: *R*^2^ = 0.98, *p* = 0.001). Meanwhile, the correlation of community structure with environments between SG1 and SG2 sharply contrasts (Figure 4A). Specifically, the community structure in SG2 had a negative correlation with Fv/Fm, NH_4_^+^, oxidation-reduction potential (ORP, mv), MAP (mm), Alt. (m), and WC (%), while had a positive correlation with PO_4_^3+^, salinity, silt, WS (m⋅s^–1^), pH, AI, MAT (°C), and MASD (h). These correlations are reversed in SG1. The results of the community structure at the family level indicated that Nitrososphaeraceae in Thaumarchaeota was dominant in SG1 (Figure 4B), followed by unclassified_d_archaea. In our study, Halococcaceae and Haloferacaceae were mainly Haloarchaea, which dominated in SG2. In summary, the abundance of various taxa varied across 200 archaeal communities. Nitrososphaeraceae dominated the archaeal communities in SG1, and their distribution pattern was positively associated with non-salinity-related environmental variables. In contrast, Haloarchaea dominated SG2, and the distribution was positively associated with salinity-related environmental variables.

### The Patterns of Community Assembly With Different Dominated Taxa

**FIGURE 5:**
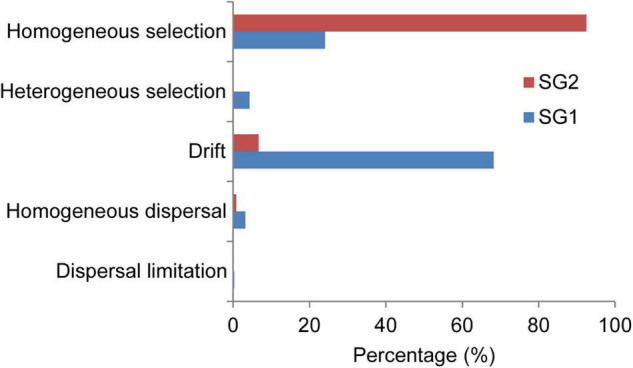
Quantification of the relative roles of ecological processes in archaeal community assembly. The percentage of each assembly process was quantified in SG1 and SG2 using Stegen’s method (Stegen et al., 2013).

We, respectively, quantified the relative roles of ecological processes in SG1 and SG2 and discovered that SG1 assembly was primarily influenced by drift alone (Figure 5), followed by a homogenous selection, while SG2 assembly was dominated by the homogeneous selection, which was significantly larger than drift. Comparatively speaking, the deterministic process had a greater impact on SG2’s community assembly than on SG1.

### The Different Co-occurrence Patterns in Two Subgroups

**FIGURE 6:**
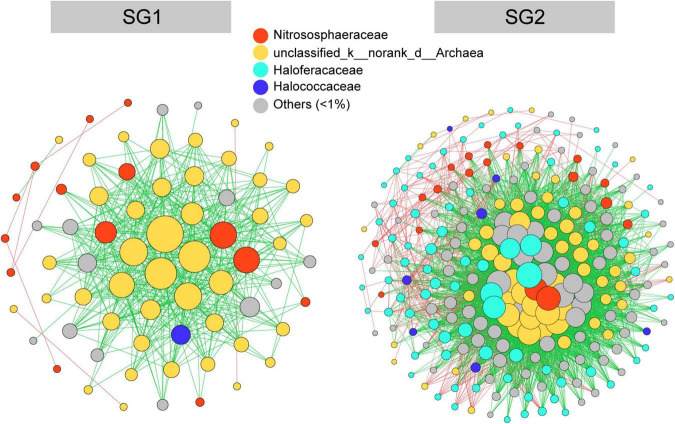
Co-occurrence network of microbiomes in SG1 and SG2. The size of each node was proportional to the degree of the operational taxonomic units. Node color was based on phylum taxa. The connection (edge) colored with red and green represented coexistence and mutual exclusion, respectively.

We derived co-occurrence networks for SG1 and SG2 (Figure 6 and Table 1). In terms of topological structure, mutual exclusion dominated the relationships of both subgroups but was stronger in SG1. However, SG2 contains more nodes, a larger number of edges per node, an average degree, and a longer average path distance than SG1. In terms of co-occurrence species, unclassified_d_archaea and Nitrososphaeraceae dominated in SG1, while Haloarchaea was barely involved. However, unclassified_d_archaea, Nitrososphaeraceae, and Haloarchaea all dominated in SG2. The relative proportion of peripheral taxa was slightly higher than that of key taxa in SG1 (55.71 vs. 44.29%), whereas the contrast was more evident in SG2 (92.46 vs. 7.54%). In summary, archaea co-occurred in a mutually exclusive pattern. The SG1 network had a higher proportion of competitions and key taxa than the SG2 network, although the network of SG2 was more complex.

**TABLE 1:** The features of the co-occurrence networks in two subgroups.

		SG1	SG2
Topological characteristics	Coexistence (%)	1.515	10.707
	Mutual exclusions (%)	98.485	89.293
	Number edges per node	6.6	17.696
	Average degree	13.2	35.357
	Average path distance	1.791	2.231
Classification of taxa	Peripheral taxa (%)	55.71	92.46
	Key taxa (%)	44.29	7.54

## Discussion

### Insights Into the Archaeal Community Differences Among Successional Stages

Many ecological effects have been suggested to understand biodiversity changes including “mass effects” and “drift,” which vary widely in importance depending on the environments (Leibold et al., 2004; Li and Hu, 2021). In particular, the effects of habitats on community heterogeneity were more emphasized in archaeal communities (Jiao et al., 2019). Our results showed that there were more macroclimate factors than microenvironments in factors that had greater explanation compared to succession factor (Supplementary Figure 1). This may result in a consequence of that the archaeal community variations in the same successional stage exceed the variations between successional stages (Figure 2B), which was quite distinct from the successional convergence of prokaryotic community in biocrusts (Xu et al., 2020). In other words, the criterion of classification for different successional stages of biocrusts based on the dominant cryptogamous plants was not applicable to the archaeal communities’ development. One possible explanation for this fact is that the archaea with low abundance in biocrusts were more vulnerable to the effects of drift (Pedros-Alio, 2006). On the other hand, archaea with unique niche were facilitated to exchange among unknown patchy habitats in the way of dispersal under the flat and windy geomorphic characteristics of northern China (Wilkinson et al., 2012). Further combining the results of biodiversity, assembly changes, and co-occurrence patterns (Figure 2), it can be inferred that archaeal communities were assembled by heterogeneous taxa driven by drift in A and M crusts, resulting in a higher α, β diversity, and mutual exclusion. In contrast, the archaeal communities in C crusts were assembled by homogeneous taxa driven by “mass effects,” which showed a low α, β diversity, and coexistence. In addition, the distributions of archaea in A and M crusts were mainly affected by microenvironments, and that in C crust were mainly controlled by macroclimates (Supplementary Table 1). This suggested that microenvironment and macroclimate variables may control the ecological processes of archaeal community assembly (Dini-Andreote et al., 2015; Zhang et al., 2016). In summary, based on the results of biodiversity, assembly, and co-occurrence patterns, we proposed that it was more suitable to classify archaeal communities by dominant taxa rather than by succession for understanding the ecological laws. Meanwhile, our results also suggested that patchy habitats played an important role in archaeal community variation.

### Spatial Factor’s Important Role in Driving Archaeal Biogeography in Biocrusts

It is crucial to determine the biogeographic patterns and their driving forces in microbial ecology. Considering that microbial community heterogeneity ranges from a few centimeters (Chu et al., 2016) to thousands of kilometers (Crowther et al., 2019), it implied that the biogeographic patterns may depend on the number of SPs considered. Commonly, the slope of factors explaining community heterogeneity with distance was used to characterize the driving force. In addition to spatial factors (Ramette and Tiedje, 2007; Martiny et al., 2011; Wang et al., 2017), it was used to examine environmental filtering effects (Chen et al., 2019; Jiao et al., 2020) and the regulation with succession (Gotelli et al., 2017). In our study, the driving force of spatial factors was greater than that of environmental and successional factors (Figure 3), and it also had the greatest explanation for archaeal community difference (Supplementary Figure 1) under the number of three to ten SPs, which could be because the number of isolated SPs of archaeal communities was higher than expected. The pronounced effects of spatial factors include not only geomorphic but also the comprehensive effects of unmeasured transient environments (Ouyang and Hu, 2017), niche conservation, dispersal rates, and historical events (Hendershot et al., 2017). The spatial effects are amplified at large scales (Kramer-Schadt et al., 2013; Li and Hu, 2021). However, another study reported that archaeal community heterogeneity was primarily driven by environments in the agricultural ecosystem (Jiao et al., 2019). The difference in driving factors could be due to the extinction of niche conservative archaea under artificial periodic interference in the agricultural ecosystem. This situation was rarely observed in dryland (Figure 3), although the strength of environmental filtering was always maintained at a high level. However, the fluctuation of macroclimates was more usual and irregular compared to the microenvironments, as indicated by the result that AI explained 48% of the total community variation (Supplementary Table 2), which sparked our interest in comparing macroclimate and microenvironment variables. Our results revealed that macroclimates explained more variation in archaeal communities than microenvironments (Supplementary Figure 2). In recent studies, succession as a factor that converged community differences has been revealed in bacterial and eukaryotic communities in biocrusts (Xu et al., 2020; Li and Hu, 2021), and comparable but lesser effects of succession have been observed in archaeal communities (Figure 3). Therefore, the ability of various microorganisms to self-organize coincided with temporal trends in the biocrust ecosystem. In summary, spatial factors were critical in improving the archaeal community heterogeneity, and succession was the only factor that converged community differences.

### The Impact of the Number of Species Pools Considered Was Non-negligible

Microbial taxa shared among habitats would be limited by their dispersal abilities and spatial distance (Liao et al., 2017; Mo et al., 2018; Li et al., 2021), and less may be shared in our sampling transect with obvious altitude gradients along the meridional direction. Therefore, we assumed that the number of isolated SPs considered could alter biogeographic patterns (Lennon and Jones, 2011; Kramer-Schadt et al., 2013). According to our results (Figure 3), successional and spatial factors drove the most apparent biogeographic patterns under the number of three SPs at the taxonomic facet, highlighting the considerable heterogeneity of archaeal assemblage composition suffering from microhabitat changes (Jiao et al., 2019, 2021). In contrast, at the phylogenetic facet, five SPs were the turning point where the slope of spatial factors began to decrease (Figure 3), and it also indicated the tipping point where the influences of community homogeneity began to emerge, which was mostly contributed by the widely distributed Nitrososphaeraceae (Figure 4A). Meanwhile, increasing homogeneity suppressed the successional driving force (Xu et al., 2020), explaining our results that weaker biogeographic patterns driven by successional factors were exhibited with an increasing number of SPs considered. Overall, the spatial driving force for taxonomic and phylogenetic variation was not synchronized with the increasing SP, but the environmental and succession driving forces were synchronized. In addition, the increasing community homogeneity from the widespread Nitrososphaeraceae suppressed the successional driving force.

### Archaeal Community Distribution Patterns Were Associated With Different Attribute Environments

Most archaea inhabit marine sediments and 8–5,000 m deep underground, accounting for approximately 20% of the total microbial community, while only 1% exists in the dryland topsoil with frequent WS changes, high light intensity, and temperature (Bates et al., 2011; Bar-On et al., 2018). According to our results, archaeal communities were dominated by Nitrososphaeraceae and Haloarchaea, whose distribution patterns were affected by non-salinity-related and salinity-related variables, respectively. Nitrososphaeraceae abundance demonstrated a negative correlation with MAT and MASD (Figure 4A), which was consistent with their non-thermophilic attribute (Tourna et al., 2011). It was also understandable that the distribution of SG1 can be explained by NH_4_ to a certain extent (Figure 4A), because NH_4_^+^ was inclined to exist as NH_3_ to participate in the ammonia oxidation process under leaning alkalinity environment of biocrusts (Yuan et al., 2019). Considering the obvious altitude gradients along the meridian of China, Alt. should contain more complicated meanings besides unmeasured moisture. In addition, MAP and WC also altered Cyanobacteria composition in dryland topsoil, affecting the organic carbon quality (Yuan et al., 2016). When this was combined with our result that Nitrososphaeraceae abundance exhibited a positive correlation with MAP and WC (Figure 4A), we concluded that Nitrososphaeraceae was associated with organic carbon quality, which implied that they could be heterotrophs. Cyanobacteria, which are also major nitrogen-fixing microorganisms in biocrusts, contribute most to Fv/Fm, a characteristic of photosynthetic capacity. The distribution of SG1 was affected by Fv/Fm (Figure 4A) implying that the ammonia oxidation process could be coupled with nitrogen fixation.

Haloarchaea is a branch of non-methanogenic archaea that thrives in salinity-dependent environments, due to a “salt-in” osmoprotection mechanism (Becker et al., 2014). This can also be proved by our results of the positive correlation between their abundance and WS, MAT, MASD, and AI (Figure 4A). Moreover, they have an adaptive evolution from anaerobic to aerobic habitats (Sorokin et al., 2017). Our results indicated that the communities of SG2 distributed along with the content of silt (Figure 4), which is easy to form anaerobic microchamber with low light (Chau et al., 2011), implying a relatively older phylogenetic position. In addition, phosphorus is not only a limiting variable of terrestrial ecosystem productivity (Cleveland et al., 2013) but also a key variable in regulating the assembly of bacterial and eukaryotic communities in biocrusts (Li and Hu, 2021). The effect of PO_4_^3+^ on the distribution of SG2 communities in this study implied that it acted as a valve in the process of community assembly and productivity allocation. The results of the mantel test also showed that there were significant relationships between SG2 and ALPT, ALP, and β-GC (Supplementary Figure 3), which suggested that Haloarchaea in biocrusts contributed to the soil carbon cycle by degrading extracellular proteins, carbohydrates, and straight-chain lipids (Iverson et al., 2012; Li et al., 2015).

### Homogeneous Selection and Drift Regulated Archaeal Community Assembly in Biocrusts

In microbial ecology, the assembly mechanism shaped biogeographic patterns. However, much remains unknown about the archaeal community assembly. To the best of our knowledge, this is the first study to reveal archaeal assembly mechanisms in dryland topsoil habitats. Our results indicated that the dominant assembly processes in SG1 and SG2 were completely different (Figure 5). First, the dynamic community assembly processes were generally regulated by environments with the greatest heterogeneity (Logares et al., 2013; Liu et al., 2020). Considering this, WC and salinity could be considered key environments for regulating assembly in SG1 and SG2, respectively (Figure 4A). Second, the spatial scale was also a non-negligible factor in altering different microorganism assembly processes. Our result indicated that stochastic processes dominated in SG1 with larger spatial scale, whereas deterministic processes dominated in SG2 with smaller spatial scale (Figure 5). This situation was consistent with the bacterial community assembly (Chen et al., 2020; Liu et al., 2020; Logares et al., 2020) but not with eukaryotic communities (Chen et al., 2017; Zhang W. et al., 2018). Third, the ability of ATPase operons to horizontally transfer expands the habitat range of Nitrososphaeraceae (Wang B. et al., 2019), which may be a more biased factor to easily detect the influence of drift based on the assembly quantitative method of phylogenetic signals. In contrast, Haloarchaea has the ability of light utilization through rhodopsin (Kamo et al., 2006), which could be restricted by the microchamber with low porosity in biocrusts considered as a deterministic factor affecting assembly. Fourth, under the same size of SP (Supplementary Figure 4), the assembly patterns of meta-communities dominated by Nitrososphaeraceae were governed by drift, while that dominated by Haloarchaea were governed by homogeneous selection, which implied that the assembly patterns of SG1 and SG2 were determined by the dominant taxa rather than the size of SPs. Collectively, the comprehensive influence of environmental, spatial, methodology, and survival strategies resulted in the different assemblies in SG1 and SG2.

### Different Co-occurrence Patterns in Subgroup1 and Subgroup2

Many studies concerning various habitats and microorganisms revealed intricate co-occurrence relationships (Liu et al., 2020; Li et al., 2021). In biocrusts, mutual exclusion dominated in archaeal communities (Figure 6), which was completely different from the high coexistence of bacterial and eukaryotic communities (Li and Hu, 2021), but was similar to archaea in river sediments resulting from the narrowing of niche breadth due to environmental filtering (Chen et al., 2020). This could be a possible explanation for the distribution patterns in our result that are related to different attributes and environmental variables (Figure 4A). Moreover, the higher proportion of homogenous microorganisms in the SG1 and SG2 (Figure 4B) resulted in microbes occupying similar resources, let alone the Nitrososphaeraceae predominate ammonia-oxidizing process in the nitrogen cycle, which enhanced the competition by simplex ecological function. Furthermore, the high proportion of key taxa in SG1 (Table 1) could contribute to the drift in the community assembly because the competition made adaptation to a new habitat more difficult. In comparison, the competition in the SG2 network was slightly weaker with less proportion of key taxa, enabling the appearance of homogeneous selection in the community assembly. In addition, the SG2 co-occurrence network had a high proportion of Haloarchaea (Figure 6), which was related to their ability to use rhodopsin for photoautotrophy (Kamo et al., 2006), and “salt-in” strategy that provided transmembrane dynamic potential in a hypersaline environment. Furthermore, in the same size of SP, the meta-communities dominated by Nitrososphaeraceae had more proportion of mutual exclusion and key taxa than that dominated by Haloarchaea (Supplementary Table 3). In summary, we believed that the dominant taxa with different survival strategies in archaeal communities determined the co-occurrence patterns and proportion of key taxa, which was also a key to understand different archaeal community assembly patterns.

## Conclusion

The archaeal community differences in the same successional stage exceed the variations between successional stages, which was due to the fact that the heterogeneous taxa tended to exchange between unknown patches driven by drift. Faced with such a great heterogeneity, we proposed that it was more suitable to classify archaeal communities by dominant taxa rather than by succession for a better understanding of the ecological laws. The distribution pattern of SG1 dominated by Nitrososphaeraceae was associated with non-salinity-related variables, while that of SG2 dominated by Haloarchaea was associated with salinity-related variables. The ecological process showed that the drift shaped the SG1 community assembly, whereas homogeneous selection played crucial roles in SG2 community assembly. The positive driving force of spatial factors and the negative driving force of successional factor were detected at both facets of taxonomic and phylogenetic variation. Furthermore, the results indicated that the impact of spatial factors on archaeal biogeography was greater than that of environmental and successional factors, both at the taxonomic and phylogenetic facets in the number of three to ten SPs. Meanwhile, the macroclimates with a stronger effect on the archaeal community variation than microenvironments were also emphasized. Finally, network analysis revealed that mutual exclusions were strong in both SG1 and SG2, while the proportion of key taxa involved in SG1 was much larger than that in SG2. Overall, these findings provided novel insights into archaeal biogeography and driving factors, especially the influences of the number of SPs, and shed new light on the distinct distribution patterns resulting from different dominated taxa assembly and co-occurrence patterns in dryland surface soil.

## Data Availability Statement

The raw sequence data presented in the study are deposited in the NCBI repository, accession number PRJNA730649. The environmental dataset can be publicly accessed on FigShare (https://doi.org/10.6084/m9.figshare.13172411.v1). They are also available from the corresponding author on reasonable request.

## Author Contributions

YL: conceptualization, methodology, investigation, formal analysis, visualization, and writing—original draft. JW: methodology. HY and DZ: investigation. CH: project administration, supervision, and writing—review and editing. All authors contributed to the article and approved the submitted version.

## Conflict of Interest

The authors declare that the research was conducted in the absence of any commercial or financial relationships that could be construed as a potential conflict of interest.

## Publisher’s Note

All claims expressed in this article are solely those of the authors and do not necessarily represent those of their affiliated organizations, or those of the publisher, the editors and the reviewers. Any product that may be evaluated in this article, or claim that may be made by its manufacturer, is not guaranteed or endorsed by the publisher.

## Footnotes

1
http://www.iqtree.org/

2
http://ieg4.rccc.ou.edu/mena

3
http://www.r-project.org/
