# Supplementary material for: Biogeographic, Driving Factors, Assembly, and Co-occurrence Patterns of Archaeal Community in Biocrusts
Source: Front Microbiol. 2022 Apr 12;13:848908. doi: 10.3389/fmicb.2022.848908 (PMC9042396; doi:10.3389/fmicb.2022.848908)
Supplement: Supplementary file 1 [file Data_Sheet_1.docx]

**ORIGINAL ARTICLE**

**Biogeography, Driving Factors, Assembly, and Co-occurrence Patterns of Archaeal Community in Biocrusts**

Author Names: Yuanlong Li^1,2^, Jingyi Wei^1,2^, Haijian Yang^1^, Delu Zhang^1^, Chunxiang Hu^1 *^

1 Key Laboratory of Algal Biology, Institute of Hydrobiology, Chinese Academy of Sciences, Wuhan, 430072, China.

2 University of Chinese Academy of Sciences, Beijing, 100049, China.

**Running title:** Archaeal biogeography, assembly and co-occurrence

^*^ Corresponding author: Chunxiang Hu

Key Laboratory of Algal Biology, Institute of Hydrobiology, Chinese Academy of Sciences (CAS), Wuhan 430072, China

E-mail: cxhu@ihb.ac.cn

Phone/Fax: +86-27-68780866


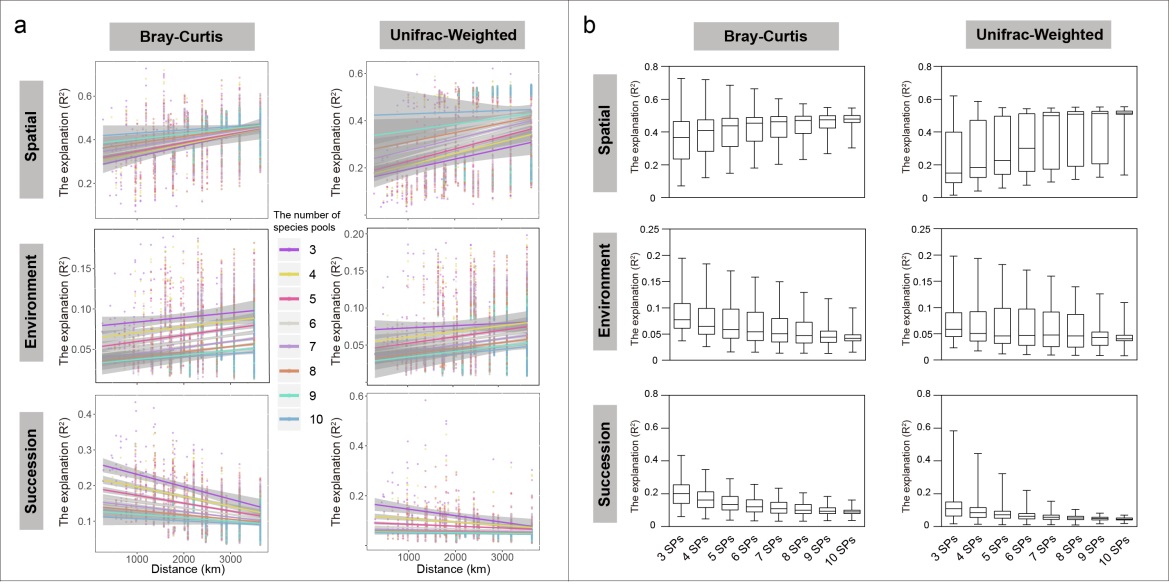


Fig. S1. The explanation of spatial, environmental, and succession factors for community variation within the various numbers of species pools (SPs). The permutational multivariate analysis of variance (PERMANOVA, permutation = 999) was used to calculate the explanations (R^2^) of successional stages (i.e. successional factor), geographical locations (i.e. spatial factor), and each of the environmental variables (i.e. environmental factor) for each combined community variation based Bray-Curtis and Unifrac-Weighted dissimilarity. The number of sample plots contained in the combined community was considered the number of SPs. (a). The linear regression between explanation and distance. The gray shadow part indicated the 95% confidence interval. (b). The explanation varied with the number of SPs.


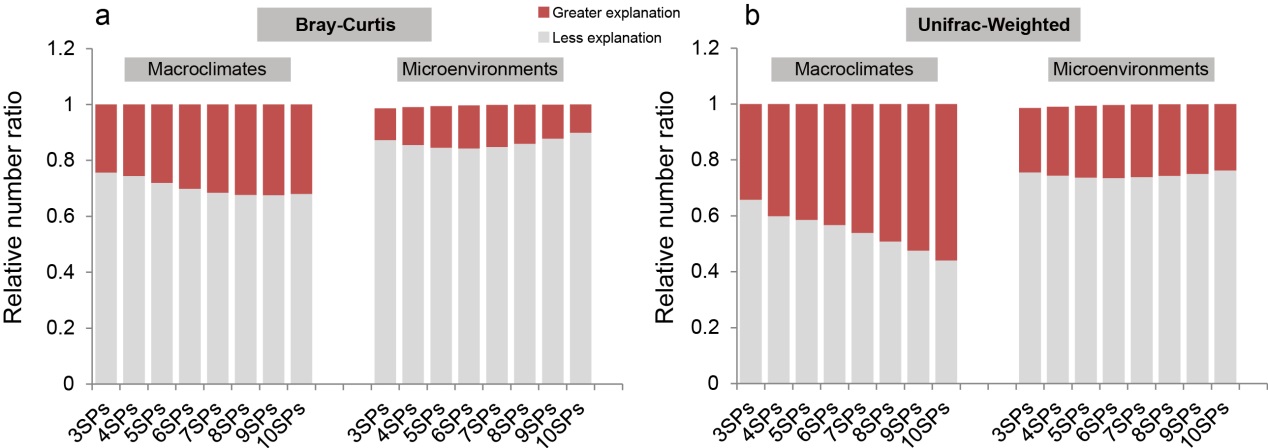


Fig. S2. The relative number ratio of macroclimates and microenvironments with greater and less explanation compared to succession factor in the various numbers of species pools (SPs). This result was derived from the results of explanation of environmental and successional factors for community variation in various numbers of SPs based on Bray-Curtis (a) and Unifrac-Weighted (b) dissimilarity. All environmental variables could be divided into macroclimates and microenvironments (as described in the method). The relative number ratio of macroclimates and microenvironments that had greater or less explanations than that of successional factors were calculated.


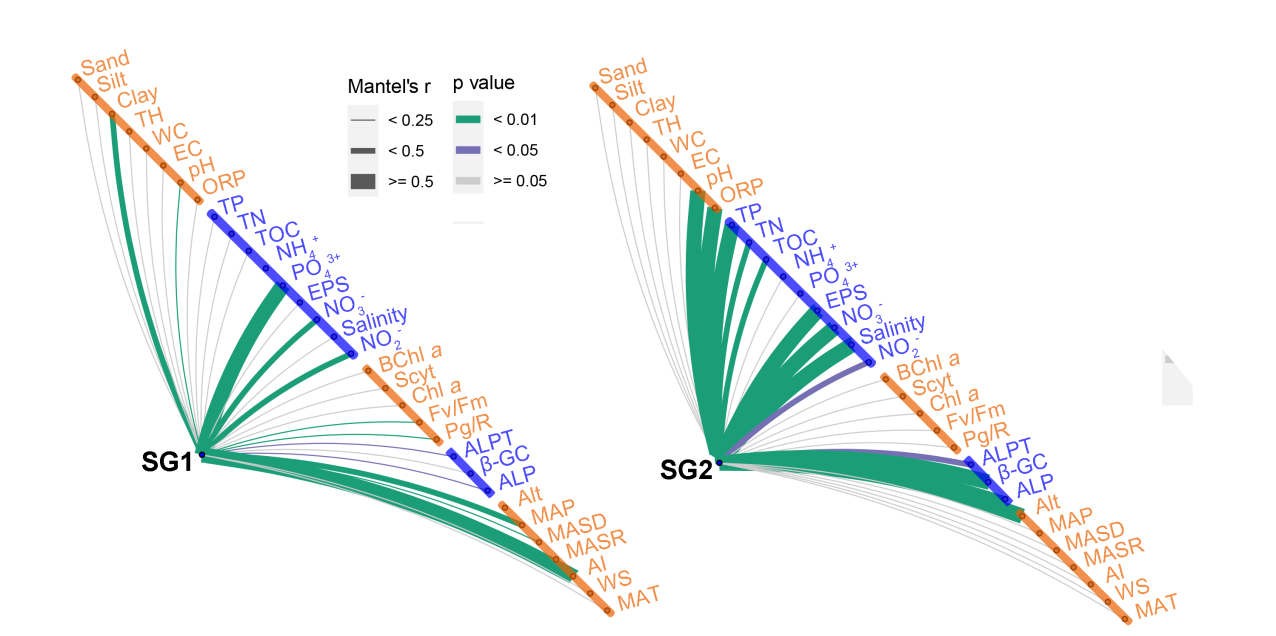


Fig. S3. Correlations between environmental factors and microbiome community at family level by Mantel test. The environmental factors were divided into five categories: soil properties (sand, silt, clay, thickness (TH.), water content (WC), pH, oxidation-reduction potential (ORP)), nutrition and ions (total phosphorus (TP), total nitrogen (TN), total organic carbon (TOC), NH_4_^+^, PO_4_^3+^, extracellular polysaccharide (EPS), NO_3_^−^, salinity, NO_2_^−^), photosynthesis and pigments (bacteriochlorophyll *a* (BChl *a*), scytonemin (Scyt.), chlorophyll *a* (Chl *a*), variable fluorescence/maximal fluorescence (Fv/Fm), Pg/R), extracellular enzyme activity (soil alkaline protease (ALPT), and soil-β-glucosidase (β-GC), soil alkaline phosphatase (ALP)), and microclimate (altitude (Alt.), mean annual precipitation (MAP), mean annual sunshine duration (MASD), aridity index (AI), windspeed (WS), mean annual temperature (MAT)), separated by yellow and blue.


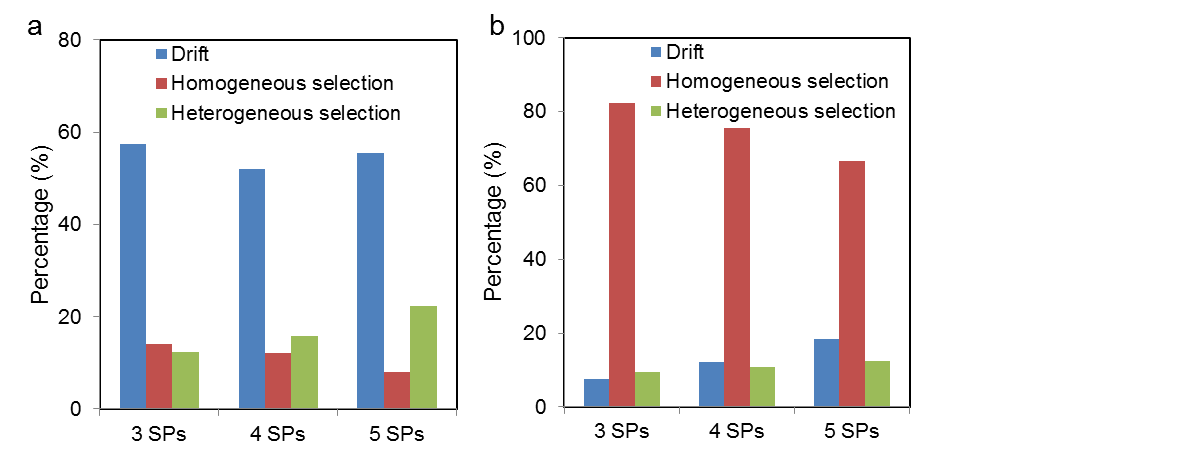


Fig. S4. The assembly patterns of meta-communities dominated by Nitrososphaeraceae and Haloarchaea in different numbers of species pools. The meta-communities which were dominated (>60%) by Nitrososphaeraceae (a) and Haloarchaea (b) were respectively selected from the 3-5 SPs to calculate the assembly processes. The values of assembly processes were shown as mean in one species pool.

Table S1. Linear regression between NMDS1 and environmental factors. Linear regression (*p*, * < 0.05, ** < 0.01) was used to study the relationship between the NMDS1 (Non-metric multidimensional scaling) based on Bray-Curtis dissimilarity at OTU level and environments in algal (A), Cyanobacterial-lichen (C), and moss (M) crusts. The environments were transformed by standardization. The values in the table showed the goodness of fit of the top 5 environmental factors. The environments with and without gray background indicated microenvironments and macroclimates, respectively.

| A crusts | |  | | | C crusts | |  | | | M crusts | |
| --- | --- | --- | --- | --- | --- | --- | --- | --- | --- | --- | --- |
| Na^+^ | 0.6244** | |  | MASR | | 0.3688** | |  | CO_3_^2-^ | | 0.7085** |
| AI | 0.6008** | |  | MAP | | 0.3521** | |  | WC | | 0.4731** |
| Cl^-^ | 0.5757** | |  | Chl *a* | | 0.2652* | |  | Clay | | 0.4658** |
| Salinity | 0.5113** | |  | AI | | 0.2603* | |  | MAP | | 0.3944** |
| SO_4_^2-^ | 0.374** | |  | TH. | | 0.2538* | |  | K^+^ | | 0.3888** |

Table S2. The explanation of environmental variables to total archaeal community variation. The forward selection was used to select significant environments that explain community variation. This step was stopped if an insignificant alpha level (*p* value > 0.05) was reached, or if there was no model improvement seen in the variation being explained (R^2^) after adding any additional variables. Abbreviations: Aridity Index (AI), Mean Annual Precipitation (MAP), Water Content (WC), oxidation-reduction Potential (ORP), Wind Speed (WS), Mean Annual Temperature (MAT), Mean Annual Sunshine Duration (MASD) and Altitude (Alt.).

| Name | Explanation (%) | Contribution (%) | pseudo-F | *p* |
| --- | --- | --- | --- | --- |
| AI | 48 | 66.9 | 183 | 0.002 |
| Salinity | 8.4 | 11.7 | 38 | 0.002 |
| PO_4_^3+^ | 3.1 | 4.3 | 15.6 | 0.002 |
| MAP | 1.8 | 2.6 | 8.6 | 0.002 |
| WC | 1.7 | 2.4 | 9.2 | 0.002 |
| ORP | 1.4 | 1.9 | 7.4 | 0.002 |
| pH | 1.1 | 1.5 | 6.1 | 0.002 |
| WS | 0.9 | 1.3 | 5.3 | 0.002 |
| Fv/Fm | 0.9 | 1.2 | 5.2 | 0.002 |
| Silt | 0.6 | 0.8 | 3.5 | 0.01 |
| NH_4_^+^ | 0.6 | 0.9 | 3.8 | 0.006 |
| MAT | 0.6 | 0.8 | 3.5 | 0.004 |
| MASD | 0.4 | 0.6 | 2.6 | 0.028 |
| Alt. | 0.4 | 0.6 | 2.5 | 0.036 |

Table S3. The features of the co-occurrence networks in meta-communities dominated by Nitrososphaeraceae and Haloarchaea. The metacommunity co-occurrence network for the certain number of species pools (SPs) using OTUs detected in at least 40% of the sample points, and captured associations based on Spearman correlation relationships with threshold of 0.6. The values of network features were demonstrated as mean.

|  |  | Nitrososphaeraceae domination | | |  | Haloarchaea  domination | | |
| --- | --- | --- | --- | --- | --- | --- | --- | --- |
|  |  | **3 SPs** | **4 SPs** | **5 SPs** |  | **3 SPs** | **4 SPs** | **5 SPs** |
| Topological features | Coexistence (%) | 2.689 | 5.697 | 6.648 |  | 12.571 | 9.665 | 10.356 |
|  | Mutual exclusions (%) | 97.311 | 94.303 | 93.352 |  | 87.429 | 90.335 | 89.644 |
|  | Edge Numbers per node | 6.6 | 6.8 | 6.3 |  | 13.661 | 18.541 | 17.623 |
|  | Average degree | 13.2 | 13.8 | 11.56 |  | 28.147 | 34.542 | 35.361 |
|  | Average path distance | 1.791 | 1.655 | 1.432 |  | 3.153 | 3.582 | 2.269 |
|  |  |  |  |  |  |  |  |  |
| Classification of taxa | Peripheral taxa (%) | 55.71 | 63.52 | 66.52 |  | 87.12 | 85.64 | 70.39 |
|  | Key taxa (%) | 44.29 | 36.48 | 33.48 |  | 12.88 | 14.36 | 29.61 |
